# Endoscopic Retrieval of a Migrated Intragastric Balloon Causing Small Bowel Obstruction: A Case Report and Review of Management Strategies

**DOI:** 10.7759/cureus.81808

**Published:** 2025-04-06

**Authors:** Loai Azar, Kai Tey

**Affiliations:** 1 Internal Medicine, Banner - University Medical Center Tucson, Tucson, USA; 2 Gastroenterology and Hepatology, University of Arizona College of Medicine - Tucson, Tucson, USA

**Keywords:** bariatric medicine, deflated intragastric balloon, endoscopic retrieval, intragastric balloon, intragastric balloon migration, intragastric balloon removal, obesity, small bowel obstruction

## Abstract

Intragastric balloons (IGBs) are widely used as a temporary weight-loss measure to induce early satiety and delay gastric emptying. Despite their safety profile, rare complications such as migration and small bowel obstruction (SBO) may occur, particularly when follow-up for timely removal is neglected. We present a case of a 48-year-old female with a history of obesity who arrived at the emergency department complaining of abdominal pain, nausea, vomiting, and obstipation of three days duration. Imaging revealed a dilated stomach and duodenum, with a fluid-filled IGB lodged in the proximal jejunum, causing SBO. Push enteroscopy was performed using a colonoscope, and the IGB was retrieved successfully from the jejunum into the stomach and removed entirely, avoiding the need for surgery. The patient recovered without complications and was discharged the following day. This case demonstrates an innovative approach to managing SBO caused by IGB migration. While surgery is typically the preferred method for small bowel impaction, endoscopic retrieval presents a viable, less invasive option. This article highlights the importance of gastroenterology consultation in managing migrated IGBs and reviews different techniques for endoscopic retrieval. We conclude that endoscopic management of migrated IGB causing SBO should be considered a first-line approach to minimize complications and shorten hospital stays, provided a backup surgical team is available.

## Introduction

As of 2023, approximately one to two out of every five individuals in the United States are struggling with obesity, which is defined as having a body mass index (BMI) of 30 kg/m² or higher [1,2]. Obesity poses a significant health risk due to its association with various comorbidities, including diabetes, hypertension, and heart disease.

One potential intervention for weight loss is the intragastric balloon (IGB). This device, which can be filled with either saline or air, is placed endoscopically into the stomach to promote early satiety. While there is currently no consensus on the optimal timing for IGB placement, it is often used as a temporary measure for patients who are seeking a more permanent weight-loss solution [3]. Between 2016 and 2022, an average of 4,400 IGBs were placed annually [4]. 

Though generally considered safe, the use of IGBs can lead to complications, which may range from mild to severe. Mild complications can include abdominal pain, dyspepsia, nausea, vomiting, dehydration, and acid reflux. More severe complications, while rare, may involve electrolyte disturbances, small bowel obstruction (SBO), perforation, bleeding, and even death [5-8]. Meta-analyses of the efficacy and safety of IGBs have reported the incidence of intestinal obstruction associated with IGBs to range from 0.17% to 0.8% [5,9]. Notably, air-filled IGBs carry a higher migration risk and SBO than fluid-filled ones [10,11]. 

The migration of the IGB and subsequent gastrointestinal obstruction can occur at any time after the device is placed, sometimes as early as the first month; however, the risk increases significantly after six months [12]. Current recommendations suggest that IGBs should be removed within the first six to 12 months of placement. When obstructions arise due to migrated IGBs, they can occur anywhere along the gastrointestinal tract, as far as the sigmoid colon [12]. 

The conventional treatment for SBO caused by an IGB is surgical intervention [12]. Meanwhile, endoscopic retrieval is primarily limited to proximal SBO in the first or second parts of the duodenum. In this report, we present a case involving a migrated gastric balloon that moved into the jejunum, resulting in a high-grade SBO. Remarkably, this balloon was successfully retrieved endoscopically. Although there have been reports of endoscopic retrieval of partially deflated IGBs causing SBO at the duodenal level, to our knowledge, this represents only the second documented case in the literature of successful endoscopic retrieval of an IGB lodged distal to the duodenum, specifically at the level of the proximal jejunum [13].

## Case presentation

**Figure 1:**
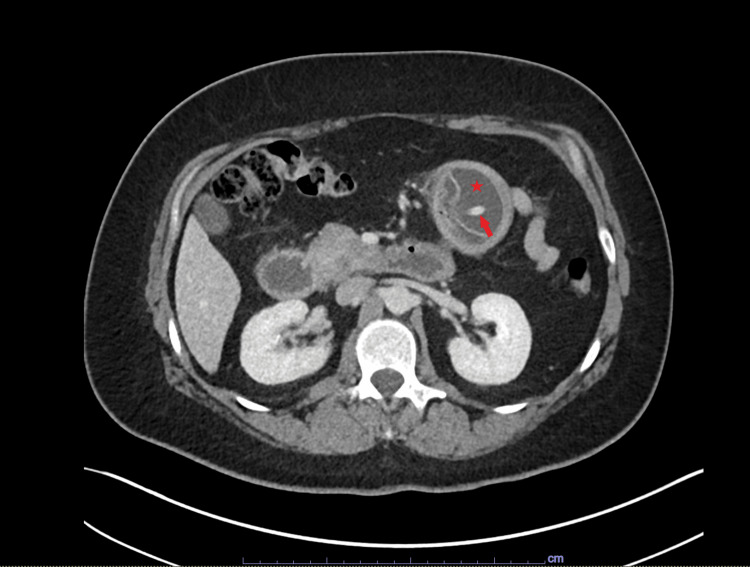
Cross-sectional computed tomography image demonstrating a device with a catheter, with a surrounding fluid-filled balloon in the proximal jejunum (arrow) The arrow indicates the catheter. The star marks the fluid-filled balloon encircling the catheter.

**Figure 2:**
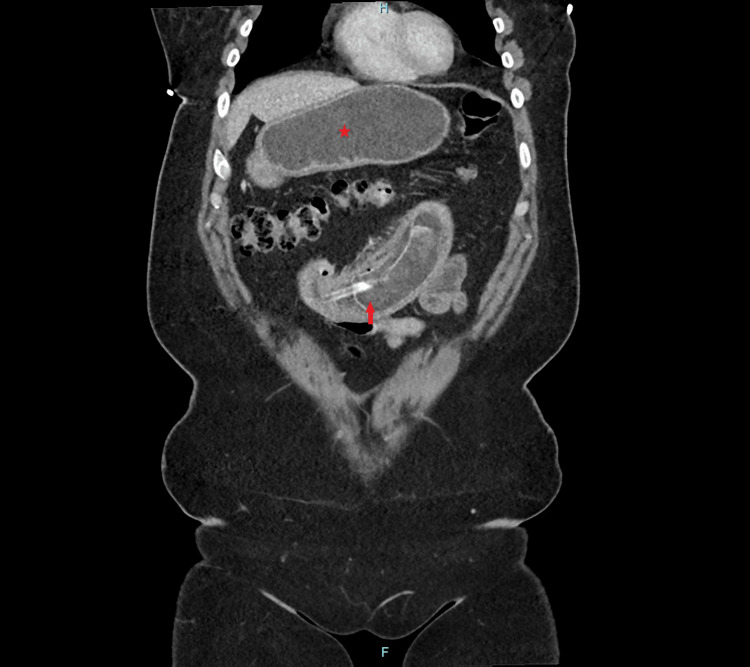
Coronal computed tomography image showing marked dilatation of the stomach, along with a device consisting of a catheter and a fluid-filled balloon in the proximal jejunum (arrow) The star indicates the distended stomach. The arrow points to the fluid-filled balloon in the proximal jejunum.

A 48-year-old female patient with a history of obesity presented to the emergency room after having an IGB inserted in Mexico two years prior, which was intended to be removed within the first year of placement. She complained of abdominal pain, nausea, vomiting, and obstipation over the past three days. Upon examination, she was vitally stable, except for mild tachycardia. Laboratory tests, including complete blood count, complete metabolic panel, and lactic acid levels, were within normal limits, though there was mild hyperglycemia and mild leukocytosis. A computed tomography scan of the abdomen revealed a dilated stomach and duodenum, as well as a fluid-filled balloon located in the proximal jejunum, causing decompression of the distal gastrointestinal tract, as seen in Figures 1, 2.

**Figure 3:**
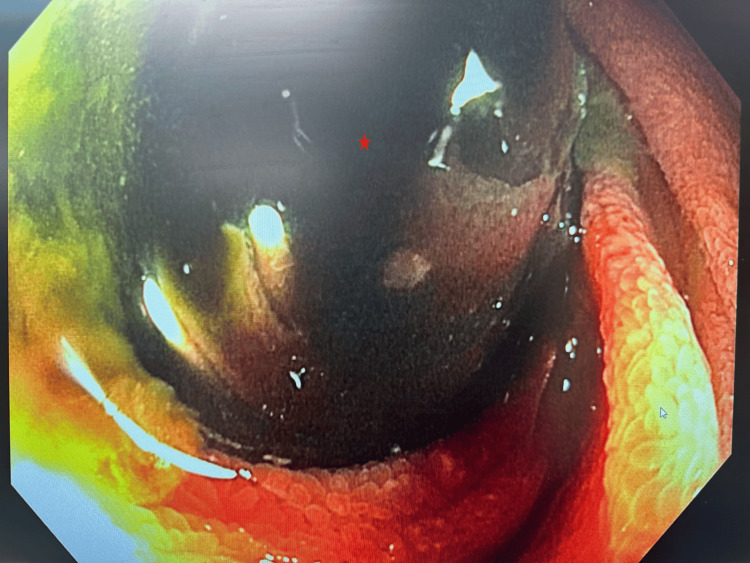
Partially inflated IGB found in the proximal part of the jejunum The star indicates the IGB in the proximal jejunum. IGB: intragastric balloon.

The patient was made NPO (nothing by mouth), and a nasogastric (NG) tube was inserted to decompress the stomach. She was admitted under the internal medicine service and taken to the endoscopy suite the following day. A pediatric colonoscope was introduced through the mouth and advanced to the proximal jejunum, allowing for small bowel enteroscopy. A foreign body was identified in the proximal jejunum, as seen in Figure 3. A rat tooth grasping forceps were used to secure the balloon, partially tearing off the balloon. Then the gastric balloon was pulled into the stomach carefully. Once repositioned, the forceps facilitated the successful removal of the balloon. The patient returned to the floor for observation and was discharged the next day.

## Discussion

Small intestinal foreign body obstruction is relatively rare, as most swallowed foreign bodies tend to become lodged in the narrowest segments of the gastrointestinal tract, specifically the oropharynx and esophagus [14,15]. However, cases of small intestinal foreign body obstruction have been documented, particularly involving a partially deflated gastric balloon that migrates distally and becomes lodged in various locations within the small intestine and rarely in the colon [5-13]. The clinical presentation generally resembles other bowel obstruction etiologies; however, a history of non-retrieval of an inserted IGB may be indicative, and imaging studies can reveal the presence of a migrated gastric balloon [5-13]. Migration can occur anytime post-insertion, although the risk is heightened after six months [5-12]. In the case discussed, the patient was advised to follow-up for gastric balloon removal within one year of insertion. 

Impaction at the small bowel can be addressed through various methods, including percutaneous needle aspiration, double balloon enterostomy, laparoscopy, or open surgery [12,16]. The success rate of minimally invasive procedures, such as endoscopy and percutaneous aspiration, varies based on the location of the obstruction and the expertise of the proceduralist. It is important to always consider the availability of backup surgical time in case the initial approach fails and a laparoscopic or open surgical intervention becomes necessary [12,16-18].

The current literature advocates surgical intervention in impaction distal to the duodenum [19]. To our knowledge, this case report is the second report documenting the successful endoscopic retrieval of a migrated IGB to the jejunum [13]. Our patient was admitted under the internal medicine service, with gastroenterology as a consultant, and general surgery was kept informed if endoscopic retrieval was unsuccessful. 

With advancements in endoscopic techniques, cases involving obstructive migrated IGBs should first be discussed with a gastroenterologist before considering surgical intervention. Endoscopic approaches are noninvasive, which can lead to shorter hospital stays and fewer complications. Furthermore, there have been reports of innovative and successful endoscopic deflation methods for managing mid-jejunum and ilial obstructions involving the deflation and subsequent retrieval of IGB, as well as colonoscopic approaches for distal ileal IGB obstructions [20-22].

## Conclusions

Small bowel obstruction due to IGBs is a rare but potentially life-threatening complication. Current guidelines recommend surgical intervention for cases of obstruction that occur distal to the duodenum. However, with advancements in endoscopic techniques, a gastroenterology consult is advisable before proceeding with surgery.

Endoscopy offers a less invasive strategy than surgical intervention. There have been successful reports of retrieving migrated IGBs located distal to the duodenum, as well as innovative endoscopic techniques such as endoscopic deflation and the use of colonoscopy for addressing distal small bowel obstructions.
